# Thermo-Responsive Starch-g-(PAM-co-PNIPAM): Controlled Synthesis and Effect of Molecular Components on Solution Rheology

**DOI:** 10.3390/polym10010092

**Published:** 2018-01-19

**Authors:** Yifei Fan, Nadia Boulif, Francesco Picchioni

**Affiliations:** Engineering and Technology Institute Groningen, University of Groningen, Nijenborgh 4, 9747AG Groningen, The Netherlands; y.fan@rug.nl (Y.F.); n.boulif@student.rug.nl (N.B.)

**Keywords:** Cu^0^-mediated living radical polymerization 1, thermo-responsive 2, starch copolymer 3, *N*-isopropylacrylamide

## Abstract

A series of highly branched random copolymers of acrylamide (AM) and *N*-isopropylacrylamide (NIPAM) have been prepared from a waxy potato starch-based macroinitiator by aqueous Cu^0^-mediated living radical polymerization (Cu^0^-mediated LRP). The NIPAM intake in the copolymer was varied between 0% and 50 mol % to evaluate the influence of chain composition on the aqueous rheological properties as well as their low critical solution temperature (LCST). The viscosity of the copolymer was found to increase with the NIPAM intake and an LCST can be observed when the NIPAM content is high enough (e.g., 50 mol %). In addition, thermo-thickening behavior was observed at a low shear rate (γ ≤ 10 s^−1^) and higher NIPAM content was found to shift the onset of thermo-thickening behavior to a lower temperature. However, the absolute increase in viscosity values is reduced with the NIPAM intake. Besides this, an interesting significant thermo-thickening behavior was also observed on highly branched starch-g-polyacrylamide at high temperatures (>80 °C), which has not been previously reported. Rheology tests also revealed a good salt-resistant property in copolymers with low NIPAM content (e.g., <25 mol %). Considering the viscosity profile in saline as compared to that in pure water, this NIPAM intake seems to represent an optimum balance of viscosity and salt-resistance performance.

## 1. Introduction

Stimuli-responsive polymers have been of great interest in the past decades due to their responsive abilities to a variety of factors, like pH [1], temperature [2], ultrasound [3], light [4], and electric/magnetic fields [5,6]. Among these polymers, thermo-responsive (or thermo-sensitive) ones are still the most widely studied subgroup since the earliest report on poly(*N*-isopropylacrylamide) (PNIPAM) [7]. According to the response of solubility to the change of temperature, generally, two different types of thermo-responsive polymer can be distinguished. One type is represented by polymers, like polyzwitterions and poly(ethylene oxide), which have an upper critical solution temperature (UCST). This means that their solubility in a given solvent increases with temperature above a given critical value, i.e., the UCST [8]. The other type comprises polymers like PNIPAM that display the opposite responsive behavior, i.e., the solubility decreases with temperature below a given critical value (LCST). Since the discovery of thermo-responsive polymers, application fields have been expanded to a variety of subjects: drug delivery [3,9], bioengineering [2,10], sensors [11,12], functional coatings [13,14], and enhanced oil recovery (EOR) [15,16].

In the above-mentioned applications, most of the polymers are supposed to perform their thermo-responsive functions in the range of 20 °C to 50 °C [2,3,4,14,17,18]. As the phase transition temperature possessed by linear PNIPAM is in the range of 31 °C to 33 °C (these values being irrespective of the polymer concentration, though slightly affected by the average molecular weight) [16,19], which falls within the described application windows’ (e.g., drug carrier and functional coating) temperature range [2,3,14], the synthesis of PNIPAM (co)polymers has been an attractive subject since the first report about its LCST phenomenon in 1968 [20]. From then on, many investigations have focused on manipulating the LCST window of PNIAPM. To achieve this, several factors should be considered including the molecular weight [19], properties of end groups [19,21], and chemical structure (molecular composition and architecture) [16,22]. Thanks to the development of controlled polymerization techniques (Atom-transfer radical-polymerization (ATRP), Reversible addition–fragmentation chain-transfer (RAFT), etc.), these kinds of studies can be carried out in a convenient and more accurate manner.

According to previous findings on PNIPAM homopolymers, the LCST can only be tuned by the structure of the end groups when the molecular weight is low enough (degree of polymerization (DP) below 200) [19,23] or the tacticity is well controlled [24]. Thus, most of the efforts have been spent on the synthesis of PNIPAM (block, random, graft) copolymers and polymers with differing architecture [22,25]. For these polymers, not only do the composition and architecture affect the LCST, but their solution rheological behavior can also be manipulated due to the inter-/intra-molecular thermo-reversible aggregation of the hydrophobic NIPAM moieties above the LCST. Although varieties of PNIPAM copolymers with different architecture have been synthesized, relatively few reports focus on their thermo-thickening properties. To the best of our knowledge, only block and graft PNIPAM copolymers have been studied so far [15,16,26,27].

In the case of grafted comb-like polymers, compared with side chains with a block structure, it has been reported that random PNIPAM copolymer side chains could endow the product with better thermo-thickening behavior (above 50 °C) and solubility in water for applications like EOR [16,28]. From this point of view, the synthesis of branched PNIPAM copolymers will be helpful to fully understanding the influence of the structure on polymers’ properties. In this case, waxy potato starch, which contains more than 95% amylopectin with a highly branched structure that is composed of 10^5^–10^6^ anhydroglucose units (AGU), will be an interesting candidate as the core due to its inherent high molecular weight and highly branched structure (see also Figure S1 illustration and Graphical abstract) [29,30,31,32].

Based on this, in the present work, random copolymers of acrylamide and *N*-isopropylacrylamide were grafted from a waxy potato starch backbone at the molecular level by Cu^0^-mediated LRP in aqueous solution. The obtained highly branched copolymer of starch-g-poly(acrylamide-co-*N*-isopropylacrylamide) (St-g-(PAM-co-PNIPAM) was characterized by ^1^H-NMR and FTIR. The influence of chain composition on the highly branched polymers’ rheological properties (at both room temperature and high temperature), LCST, and their response to salinity was studied and compared with the comb-like copolymer reported [16].

## 2. Materials and Methods

### 2.1. Materials

Waxy potato starch (>95% amylopectin, molecular weight in the range 10^7^–10^9^ Da and roughly 5% of α (1–6) branching points) was kindly donated by Avebe (Veendam, The Netherlands) and dried under vacuum at 60 °C for 48 h before use. Lithium chloride was purchased from Sigma-Aldrich and dried under vacuum at 80 °C for 24 h before use. Anhydrous *N*,*N*-dimethylacetamide (DMAc) was purchased from Sigma-Aldrich in Sure/Seal™ (Steinheim, Germany). 2-bromopropionyl bromide (BpB), formaldehyde solution (37%), and formic acid (>95%) were purchased from Sigma-Aldrich and used as received. Tris(2-aminoethyl)amine (Tren) was purchased from Tokyo Chemical Industry Co., Ltd. (TCI, Tokyo, Japan) and used as received. Tris[2-(dimethylamino)ethyl]amine (Me_6_Tren) was synthesized following the procedures reported [33]. *N*-Isopropylacrylamide (NIPAM, stabilized with 4-Methoxyphenol (MEHQ)) was purchased from TCI and recrystallized from acetone to remove the inhibitor. Acrylamide (AM) was purchased from Sigma-Aldrich and used as received. Copper powder (<75 μm) was purchased from Sigma-Aldrich and stored under an N_2_ atmosphere.

### 2.2. Characterization

NMR spectra were recorded on a Varian Mercury Plus 400 MHz spectrometer (Varian, Inc., Palo Alto, CA, USA) using deuterated solvents purchased from Sigma-Aldrich. Fourier Transform Infrared (FTIR) spectra were recorded with attenuated total reflection (ATR) accessories on an IRTracer-100 SHIMADZU Fourier Transform Infrared Spectrophotometer (Shimadzu Corp., Kyoto, Japan) and data were processed with LabSolutions IR software (Version 2.11, Shimadzu, Kyoto, Japan, 2014). Aqueous gel permeation chromatography (GPC) was conducted on an Agilent 1200 system (Agilent, Santa Clara, CA, USA) equipped with a differential refractive index (DRI) detector and column set (PSS SUPREMA 100 Å, 1000 Å, 3000 Å) from Polymer Standard Service GmbH (PSS, Mainz, Germany). The mobile phase used was 0.05 M NaNO_3_. Column oven and detector temperatures were regulated to 40 °C, with a flow rate of 1 mL/min. Polyacrylamide standards from PSS were used for calibration. Samples were filtered through a membrane with 0.22 μm pore size before injection. Experimental molar mass and polydispersity index (PDI) values of synthesized polymers were determined by conventional calibration using PSS WinGPC UniChrom GPC/SEC software (Version 8.20, Polymer Standards Service GmbH, Mainz, Germany, 1992–2014).

Rheological properties were measured using a HAAKE Mars III (Thermo Scientific, Waltham, MA, USA) rheometer equipped with a cone-and-plate geometry (diameter 60 mm, angle 2°). Solution viscosity was measured as a function of shear rate (0.1 to 1750 s^−1^, T = 20 °C), salt concentration (5000~100,000 ppm of NaCl, T = 20 °C, shear rate 10 s^−1^) and temperature (10 °C to 90 °C, shear rate 1 s^−1^, 3 s^−1^, 10 s^−1^ and 30 s^−1^), respectively.

The intrinsic viscosity was determined according to the Martin equation [34]:

(1) $$\eta_{red}=\frac{\eta_{sp}}{c}=\left[\eta \right]e^{k_{M}c\left[\eta \right]}$$

where $\eta_{red}$ is the reduced viscosity, $\eta_{sp}$ is the specific viscosity, *c* is the polymer concentration, [*η*] is the intrinsic viscosity, and $k_{M}$. is a constant dependent on the polymer–solvent system.

The relaxation time ( λ ) was determined according to the “Carreau–Yasuda” model [35,36,37]:

(2) $$\frac{\eta -\eta_{\infty }}{\eta_{0}-\eta_{\infty }}\;={\left[1+{\left(\lambda \cdot \gamma \right)}^{\alpha }\right]}^{\frac{n-1}{\alpha }}$$

where *η* is the viscosity, $\eta_{0}$ is the zero shear rate viscosity, $\eta_{\infty }$ is the viscosity at the infinite shear rate, 1/λ is the critical shear rate for the onset of shear thinning, *n* − 1 is the power law index, and  α represents the transition region between $\eta_{0}$ and the power law region.

The cloud point of the different polymers was determined by UV–vis analysis. A JASCO V-730 UV–vis spectrophotometer (JASCO, Easton, MD, USA) equipped with a temperature-controlled six-position sample holder was used. The transmittance of the polymer solutions (1.2 wt %) was recorded at 350 nm at temperature ranges from 20 °C to 95 °C against a reference sample containing demineralized water. Temperature was manually controlled with the software, and each measurement was taken after the temperature was stabilized within ±0.5 °C for 30 s.

### 2.3. Synthesis of Starch-Based Macroinitiator (StBr)

Waxy potato starch (2.59 g, 16 mmol) and lithium chloride (1.02 g, 24 mmol) were added to a 250 mL three-necked flask (dried overnight at 100 °C before use) connected with a mechanical stirrer. The system was vacuumed under heat and backfilled with N_2_ three times to remove residual water. Anhydrous DMAc (100 mL) was transferred to the flask and the mixture was stirred at 130 °C for about 1 h under an N_2_ atmosphere. A transparent solution formed when the mixture cooled down to room temperature naturally. The solution was cooled down with an ice bath and then 0.42 mL (4 mmol) BpB was added dropwise within 30 min under the protection of N_2_. The mixture was then warmed up naturally to room temperature and stirred for 3 h. The final products were precipitated out with tenfold acetone and then filtered, washed, and dried under vacuum at 45 °C for 24 h. The resulting white powder was then purified by Soxhlet extraction with ethanol as the solvent for 24 h (final yield: 87%). The obtained degree of substitution (DS) represents a convenient compromise as it allows a proper characterization (not possible for lower values where spectroscopic data are difficult to identify), while, at the same time, does not compromise the solubility in water (for too high DS values).

### 2.4. Synthesis of St-g-(PAM-co-PNIPAM) by Aqueous Cu^0^-Mediated LRP

Typical Polymerization Protocol: H_2_O (100 mL), StBr (48.6 mg, 0.04 mmol), a mixture of AM and NIPAM (240 mmol in total), and Me_6_TREN (23 μL, 0.08 mmol) were charged to a 250 mL three-neck round-bottom flask with a magnetic stirrer bar and rubber septum. The solution was deoxygenated by three freeze–pump–thaw cycles. Cu powder (5.2 mg, 0.08 mmol) was then added with rapid stirring under the protection of nitrogen. The mixture was allowed to react for 15 min at room temperature. The resulting solution was freeze-dried and followed by Soxhlet extraction with ethanol as the solvent for 48 h. The product was then vacuum-dried at 65 °C for 48 h. For the purposes of brevity and clarity, taking the grafted product with no NIPAM content as an example, the sample was named St-g-PNIPAM-P0; 0 here stands for the fact that the mole percentage of NIPAM in the feeding AM/NIPAM monomer mixture is 0%.

### 2.5. Cleaving of Graft Polymer Chains from the Starch Backbone

The starch-based copolymer (0.25 g) was dissolved in 25 mL Milli-Q water in a round-bottom flask, and 0.25 mL concentrated hydrochloric acid was then added. The mixture was stirred and refluxed at 100 °C for 3 h. The resulting free polyacrylamide (PAM) was precipitated out with methanol, then filtered and washed with methanol three times. The product was dried under vacuum at 60 °C for 24 h.

## 3. Results and Discussion

The successful synthesis of water-soluble waxy potato starch-based macroinitiator (StBr) was proved by FTIR and NMR (^1^H-NMR, ^13^C-NMR, and gHSQC) characterization. Details can be seen in the supporting information (Figures S2 and S3). 

**Figure 1:**
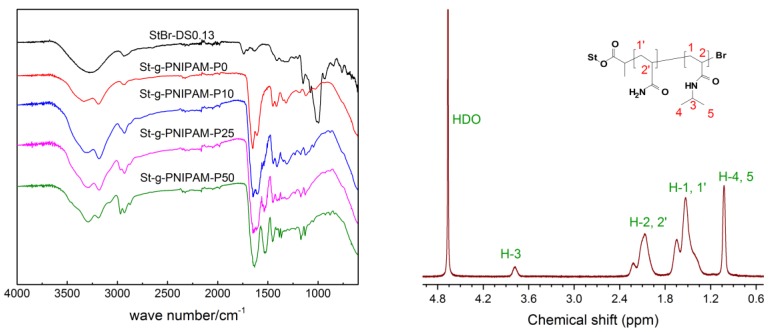
FTIR spectra of StBr and Starch-g-poly(acrylamide-co-*N*-isopropylacrylamide) (St-g-(PAM-co-PNIPAM)) with different N-isopropylacrylamide (NIPAM) mole ratio (**Left**) and ^1^H-NMR spectra of St-g-(PAM-co-PNIPAM) in D_2_O (**Right**).

A series of St-g-(PAM-co-PNIPAM) with different NIPAM molar intakes were then synthesized by Cu^0^-mediated LRP with StBr as the initiator and Cu powder/Me_6_Tren as the catalyst system. According to our previous work, the target DP for all the samples was set to 6000 to achieve satisfactory viscosity values, for example, for EOR applications. The polymer was characterized by FTIR (Figure 1, Left) and ^1^H-NMR (Figure 1, Right). The absorption peak around 3188 cm^−1^ in the FTIR spectrum was attributed to the stretch of the N–H bond in the amide group. For the St-g-PNIAPM-P0, typical amide group peaks at 1652 cm^−1^ (amide I) and 1610 cm^−1^ (amide II) could also be seen in the spectrum. With increasing NIPAM intake in the copolymer, the amide I and II peaks gradually shifted to 1628 cm^−1^ and 1530 cm^−1^, respectively [38]. The ^1^H-NMR spectrum of the copolymer is shown in Figure 1 (Right), in which the peak around 1.0 ppm was attributed to the methyl protons in the NIPAM unit. The peak at 3.8 ppm originates from the tertiary carbon protons in the NIPAM amide group. The signals in the range of 1.9–2.3 ppm and 1.2–1.8 ppm were assigned to the tertiary and secondary carbon protons in the copolymer backbone, respectively.

As shown in Table 1, the NIPAM content in the monomer mixture was varied from 0% to 50% (mol %) while the overall target DP was set to 6000 for all the polymers. ^1^H-NMR was used to determine the mole percentage of the NIPAM unit in the product according to the following equation:

(3) $$R_{NIPAM}\left(\%\right)=\frac{3}{A}\times 10$$

where *A* is the sum of the integrals of peaks H-2,2′ and H-1,1′ when the integral of peak H-3 was set to 1 (Figure 1, Right). Clearly, the composition of the resulting copolymer is quite similar to that of the feed, as shown in Table 1. This clearly suggests almost equal reactivity ratios for AM and NIPAM [39].

Due to the high polymerization rate and the corresponding high solution viscosity, it was not possible to take samples to monitor the polymerization kinetics. Nevertheless, at the end, the PAM homopolymer was thus cleaved from the St-g-PNIPAM-P0 backbone and characterized by GPC to determine whether it is a controlled polymerization. As shown in Table 1 and Figure S4, compared with the grafted starch copolymer with PDI of 2.15, a narrower molecular weight distribution (PDI = 1.36) that indicated a well-controlled polymerization was observed after the hydrolyzation of the starch backbone. It is very difficult to estimate whether all initiation sites actually contributed to the grafting of the chains (especially for the copolymers, where no GPC data after cleavage can be collected) even if it is clear that those sites that reacted resulted in polymeric chains with relatively narrow PDI values. This might stem from a different reactivity of the individual starch macromolecules and actually explain the shoulder in the GPC traces (Figure S4).

**Figure 2:**
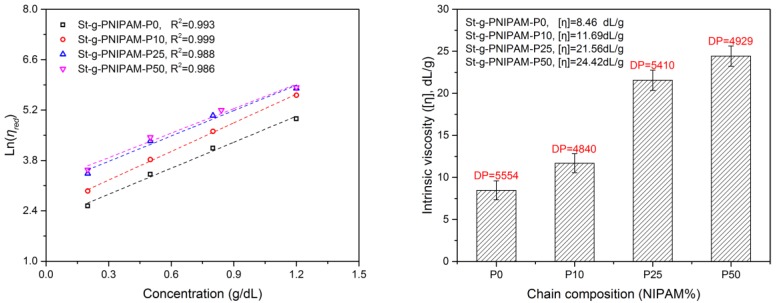
Reduced viscosity as a function of copolymer concentration (Martin’s equation) (**Left**) and corresponding intrinsic viscosity (**Right**).

To investigate the influence of chain composition on copolymer solution properties, a series of rheology tests were carried out. For a polymer with a given molecular structure, the intrinsic viscosity is an indication of polymer’s hydrodynamic volume [40,41]. The intrinsic viscosity can be obtained by extrapolating the plot of $\ln\left(\eta_{red}\right)$ against polymer concentration to *c* = 0 (see Experimental section and Figure 2).

As can be observed in Figure 2, the intrinsic viscosity increased with the NIPAM content in the grafted copolymer. Considering the chain length of PAM-co-PNIPAM, the intrinsic viscosity of St-g-PNIPAM-P25 and St-g-PNIPAM-P50 is significantly higher than that of St-g-PNIPAM-P0 and St-g-PNIPAM-P10, respectively. This means that the incorporation of the NIPAM unit expands the hydrodynamic volume of St-g-PAM in water, which can be explained by the breaking of the strong intra-hydrogen bond between AM units. This could also explain why St-g-PNIPAM-P0 has poor solubility in cold water compared with the rest of the prepared copolymers.

**Figure 3:**
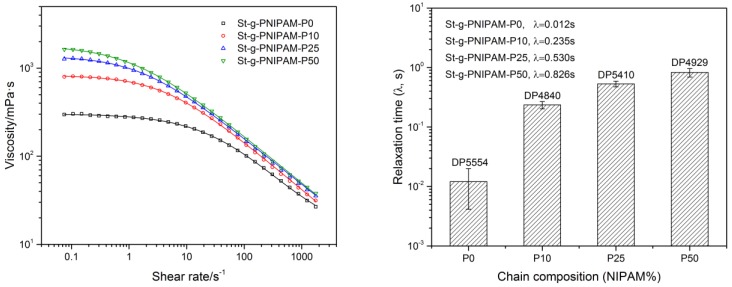
Viscosity as a function of shear rate (**Left**, 1.2 wt % copolymer solution) and corresponding relaxation time from the “Carreau–Yasuda” model (**Right**).

The influence of chain composition on solution viscosity as a function of shear rate was also evaluated at the same polymer concentration (1.2 wt %), the result of which is shown in Figure 3 (Left). It is clear that the viscosity of copolymers with a larger NIPAM content is higher than that of those with a lower NIPAM ratio in composition (at the same overall DP), especially in the low shear rate region. This was attributed to the expansion of molecular hydrodynamic volume, as indicated in the evaluation of the intrinsic viscosity. Furthermore, these data were also fitted with the “Carreau–Yasuda” model (see Experimental section) to study the influence of chain composition on the copolymer’s relaxation time (λ) (Figure 3, Right). As indicated in Figure 3 (Right), a higher NIPAM unit content leads to higher λ and, thus, a lower critical shear thinning rate (1/λ) should be observed in the flow curve. This is in line with Figure 3 (Left), which shows a shift in the onset of shear thinning behavior towards lower shear rate regions as the mole ratio of NIPAM deceases.

**Figure 4:**
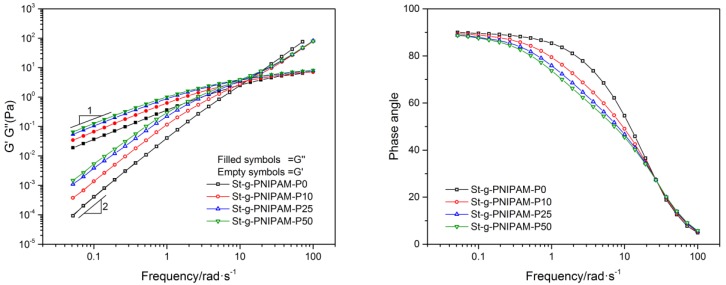
The storage (G′), loss storage (G′′) (**Left**) and phase angle (**Right**) as a function of frequency at 1.2 wt % copolymer concentration.

Besides viscosity, the viscoelastic property is another important factor that effects the applications of polymers. For example, it has been reported that higher elasticity is beneficial for improving the sweep efficiency in EOR [42,43]. The influence of composition on the copolymers’ viscoelastic properties is shown in Figure 4. As can be observed, both the storage modulus (G′) and the loss modulus (G′′) increased as the NIPAM ratio in the copolymer increased. In the terminal zone (low frequencies region), for all the samples, entangled polymer solution flow behavior with G′′ directly proportional to the frequency (ω) (slope = 1) and G′ proportional to ω^2^ (slope = 2) was indicated [44]. A comparison of phase angles (ω < 20 rad/s) at equal polymer concentrations demonstrates that the copolymer with a higher NIPAM ratio displays a more pronounced elastic response, especially when compared with that of copolymers with a similar grafted chain length (e.g., St-g-PNIPAM-P0 and St-g-PNIPAM-P10).

**Figure 5:**
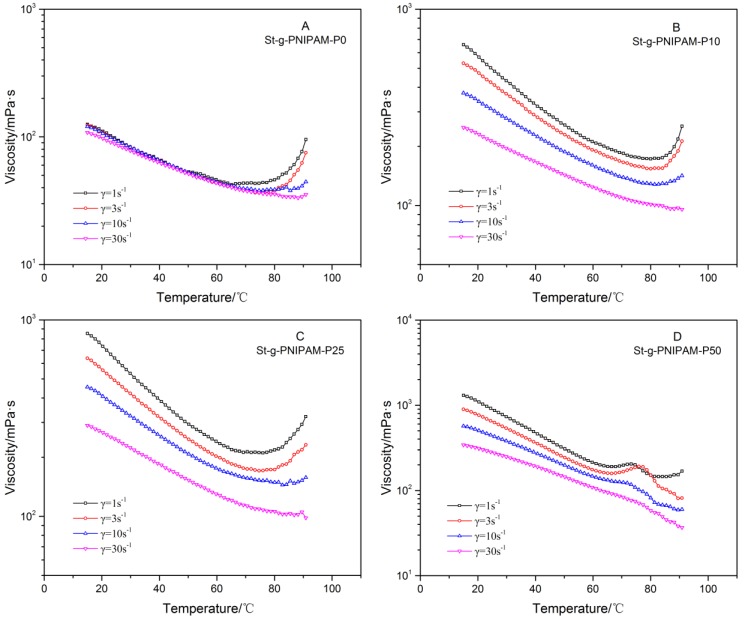
Viscosity versus temperature of (**A**) St-g-PNIPAM-P0; (**B**) St-g-PNIPAM-P10; (**C**) St-g-PNIPAM-P25; and (**D**) St-g-PNIPAM-P50 with 1.2 wt % copolymer concentration.

For NIPAM (co)polymers, the influence of structure and composition on their thermo-responsive behavior is also of great interest for applications. The viscosity (solutions with the same polymer concentrations) as a function of temperature was measured at different shear rates, and the results are displayed in Figure 5.

Linear PAM is not a thermo-responsive polymer in solution and neither is PAM with the comb-like structure according to previous reports [16,35]. However, an interesting thermo-thickening behavior was observed for the starch-based highly branched PAM when the temperature is above 80 °C (Figure 5A), especially at a low shear rate. During measurements, the cone and plate were covered with a cap to avoid water evaporation. Water evaporation as a cause for the increase in viscosity can be ruled out also based on the fact that this was not observed for linear PAM (M_n_ = 35,200) (see Figure S5). Considering the lower viscosity at room temperature compared with copolymers containing NIPAM (vide supra), this thermo-responsive behavior was attributed to the breakdown of strong intra-molecular hydrogen bonds at high temperatures, which is beneficial for the expansion of hydrodynamic volume. At a high shear rate, the inter-molecular interaction (physical entanglement, hydrogen bonding, and hydrophobic association in the case of the NIPAM copolymer) is broken down so that the thermo-thickening effect is suppressed. Thermo-responsive polymers are of interest for many applications. For example, in EOR, this shear-sensitive thermo-thickening behavior is favorable because it endows the solution with a relatively low viscosity (in turn, beneficial for energy saving) in a wellbore due to the high flow rate (thus, high shear rate). In a reservoir, however, higher viscosity will be displayed because of the low shear rate resulting from the porous structure of the oil formation—in turn, generating elastic instabilities and turbulent flow. Figure 5 also reveals that when NIPAM is copolymerized with acrylamide, the intra-molecular hydrogen bonding is inherently weakened and, thus, the incremental ratio of viscosity from the onset of thermal-thickening decreases at higher NIPAM ratios. Further, comparing the viscosity profiles at the shear rate of 1 s^−1^, we notice that the onset of thermal-thickening shifted to a lower temperature gradually as the ratio of NIPAM increased in the copolymer.

**Figure 6:**
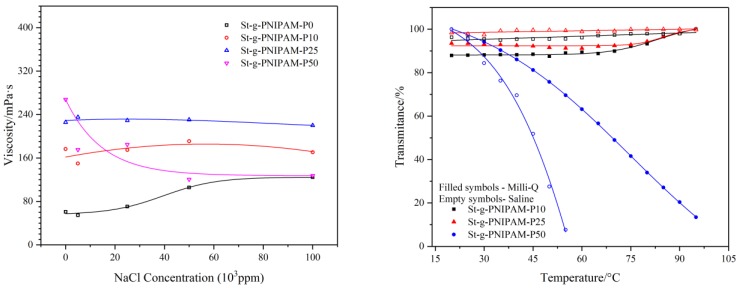
Influence of composition and salt concentration on polymers’ viscosity (γ = 10 s^−1^) (**Left**) and cloud point (**Right**) with 1 wt % copolymer solution.

Not only temperature effects the performance of NIPAM (co)polymers; salinity also has an impact on their solution properties. The viscosity as a function of salt (NaCl) concentration was measured at a shear rate of 10 s^−1^. The result is shown in Figure 6 (Left). Besides this, the influence of salt (0 ppm and 100,000 ppm) on the copolymer’s cloud point was also studied and the result is shown in Figure 6 (Right).

As can be seen in Figure 6, an increasing trend in the viscosity of St-g-PNIPAM-P0 was implied as the salt concentration increased. This could be explained by the “structure” change of water (e.g., the decrease of “free water”) due to the addition of salt [45]. Contrasting with the behavior of St-g-PNIPAM-P0, copolymers containing NIPAM in a low mole ratio (10 mol % and 25 mol % in this case) displayed a relatively stable viscosity profile versus salt concentration. This is very different from partially hydrolyzed polyacrylamide (HPAM), the viscosity of which normally displays an abrupt drop due to the collapse of molecular hydrodynamic volume resulting from the salt screening of the repulsive electrostatic forces between charged carboxyl groups [46]. For copolymers containing a high mole ratio of NIPAM (50% for example), a decreasing trend can be observed with the increase of salt concentration. This was blamed on the “salt out” effect of NaCl on NIPAM units in the copolymer, which caused shrinking of the polymer hydrodynamic volume and thus reduced the viscosity [47].

In the case of the cloud point, as shown in Figure 6 (Right), copolymers with a low NIPAM ratio (10 mol % and 25 mol %) displayed no phase transition in the temperature range of 20 °C to 95 °C. The addition of salt has no significant influence on their cloud point. St-g-PNIPAM-P50, however, has a cloud point at around 68 °C (50 mol % of the transmittance) due to high NIPAM content. The addition of NaCl (100,000 ppm) shifted the cloud point to 45 °C.

## 4. Conclusions

Different highly branched random copolymers of acrylamide (AM) and *N*-isopropylacrylamide (NIPAM) were prepared with a water-soluble waxy potato starch-based macroinitiator by aqueous Cu^0^-mediated living radical polymerization (Cu^0^-mediated LRP) at room temperature. The mole ratio of NIPAM was varied in the range of 0% to 50% to investigate the influence of chain composition on the polymers’ aqueous rheological properties as well as their LCST. The viscosity of the grafted copolymer was found to increase as the NIPAM ratio increased, due to the breakdown of the intra-molecular hydrogen bond. Compared with commercially available linear partially hydrolyzed polyacrylamide, the viscosity of the highly branched AM/NIPAM copolymer also displayed good stability in saline water. However, too much NIPAM content is not preferable for the copolymers’ salt-resistance performance, which can also be proved by the phase transition behavior (LCST) of the different copolymers. 

As expected, the viscosity versus temperature profile revealed the low shear rate (γ ≤ 10 s^−1^) thermo-thickening phenomenon of AM/NIPAM copolymers. Unlike the reported comb-like random copolymer [16], however, a high NIPAM ratio (50 mol %) doesn’t endow the highly branched copolymer with as significant a thermo-thickening property as that of a low NIPAM content copolymer. Surprisingly, a pronounced thermo-thickening behavior was also observed on highly branched starch-g-polyacrylamide at high temperatures (>80 °C), which has not been reported before. This was attributed to the disruption of the strong intramolecular hydrogen bonds originating from the highly branched structure at high temperatures.

Considering the cheapness of the starch, the ease of synthesis, and the copolymers’ saline resistance and thermo-thickening behavior in water, the highly branched random AM/NIPAM copolymers could be good candidates for applications like EOR.

## Figures and Tables

**Table 1:** Experimental data for St-g-(PAM-co-PNIPAM) synthesized by Cu^0^-mediated living radical polymerization (LRP).

Sample	Monomer Ratio ^a^ (AM:NIPAM)	Time/min	Conversion/% ^b^		Ratio ^c^ (NIPAM)	DP ^d^	PDI ^e^
			AM	NIPAM			
St-g-PNIPAM-P0	100:0	12	91.56	-	0	5554	1.36
St-g-PNIPAM-P10	90:10	12	80.66	80.66	10	4840	1.64
St-g-PNIPAM-P25	75:25	15	92.57	82.95	23	5410	- ^f^
St-g-PNIPAM-P50	50:50	12	87.08	77.22	47	4929	- ^f^

^a^ Overall ratio for polymerization [M]/[I]/[Cu^0^]/[L]= 6000:1:2:2; ^b^ Monomer conversion determined by mass and NMR; ^c^ NIPAM unit ratio in the copolymer, determined according to ^1^H-NMR; ^d^ Degree of polymerization (DP) determined by mass and NMR; ^e^ Polydispersity index (PDI) values for (co)polymer cleaved from the starch backbone; ^f^ Not available due to potential high intermolecular association, leading to retention of the polymer inside the gel permeation chromatography (GPC) column.

## Supplementary Materials

The following are available online at www.mdpi.com/2073-4360/10/1/92/s1, Figure S1: Illustration for the structure of amylopectin and grafted copolymer, Scheme S1: Synthesis of waxy potato starch-based ATRP macroinitiator and St-g-PAM, Figure S2: FTIR spectra of Starch-Br with different DS, Figure S3: ^1^H-NMR (a), ^13^C-NMR (b) and gHSQC (c) spectra of St-Br (DS = 0.13) in D_2_O, Figure S4: Molecular weight distribution of St-Br, St-g-PNIPAM-P0 before and after hydrolysis (St-g-PNIPAM-P0-H), Figure S5: Viscosity versus temperature of PAM (M_n_ = 35200) with 1.5 wt % copolymer concentration (γ = 1 s^−1^).
