# Supplementary material for: Thermo-Responsive Starch-g-(PAM-co-PNIPAM): Controlled Synthesis and Effect of Molecular Components on Solution Rheology
Source: Polymers (Basel). 2018 Jan 19;10(1):92. doi: 10.3390/polym10010092 (PMC6414930; doi:10.3390/polym10010092)
Supplement: Supplementary file 1 [file polymers-10-00092-s001.pdf]

polymers

Supplementary Material: Thermo-Responsive Starch-g-(PAM-co-PNIPAM): Controlled Synthesis and Effect of Molecular Component on Solution Rheology

Yifei Fan, Nadia Boulif, Francesco Picchioni

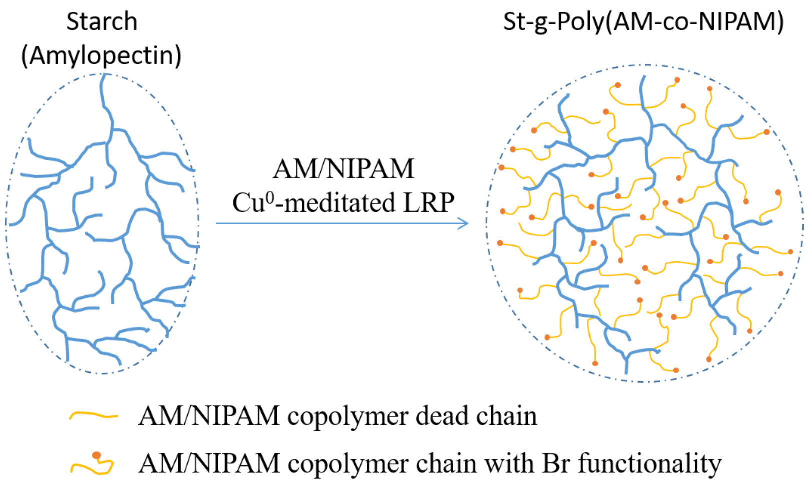

Figure S1. Illustration for the structure of amylopectin and grafted copolymer.

1. The Synthesis of Starch-Based Macroinitiator

The starch based macroinitiator StBr was prepared homogeneously via one step esterification reaction with 2-bromopropionyl bromide (BpB) in DMAc/LiCl as depicted in Scheme S1. Both FTIR (Figure S1) and NMR (Figure S2a ^{1}H-NMR, S2b ^{13}C-NMR, S2c gHSQC) were employed to demonstrate the successful preparation of the macroinitiator. The absorption peak at 1743 cm^{-1} in FTIR spectrum was assigned to the stretch of the C=O group from the initiator while the peak at 1281 cm^{-1} was attributed to the C–O bond in the ester group. The successful synthesis of StBr was further proved by NMR spectrum. In Figure S2a, the peak at 5.4 ppm should be assigned to the proton attached to the anomeric carbon and peaks in the range of 3.3–4.2 ppm should be attributed to the rest protons of the anhydroglucose unit (AGU). Peaks around 1.7 ppm and 4.7 ppm belong to methyl protons and methine proton of the 2-bromopropionyl group, respectively. The degree of esterification (DS) of StBr could be quantified with the peak at 5.4 ppm and 4.7 ppm. In the ^{13}C NMR spectrum (Figure S2b), the peak at 100 ppm belongs to the anomeric carbon and the peaks range from 60 ppm to 80 ppm should be assigned to the rest AGU carbons. The resonance of methyl and methine carbon locate at 22 ppm and 54 ppm respectively. These assignments are verified by the gHSQC spectrum (Figure S2c).

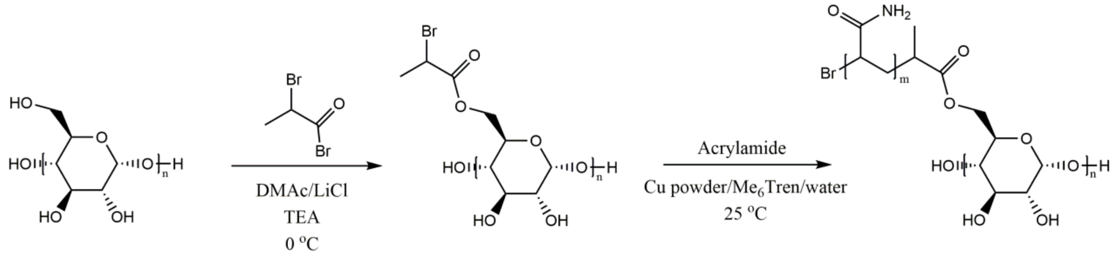

Scheme S1. Synthesis of waxy potato starch-based ATRP macroinitiator and St-g-PAM.

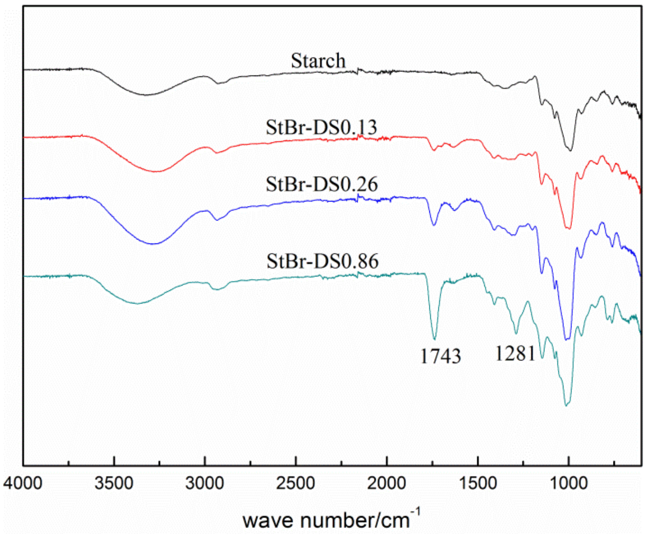

Figure S2. FTIR spectra of Starch-Br with different DS.

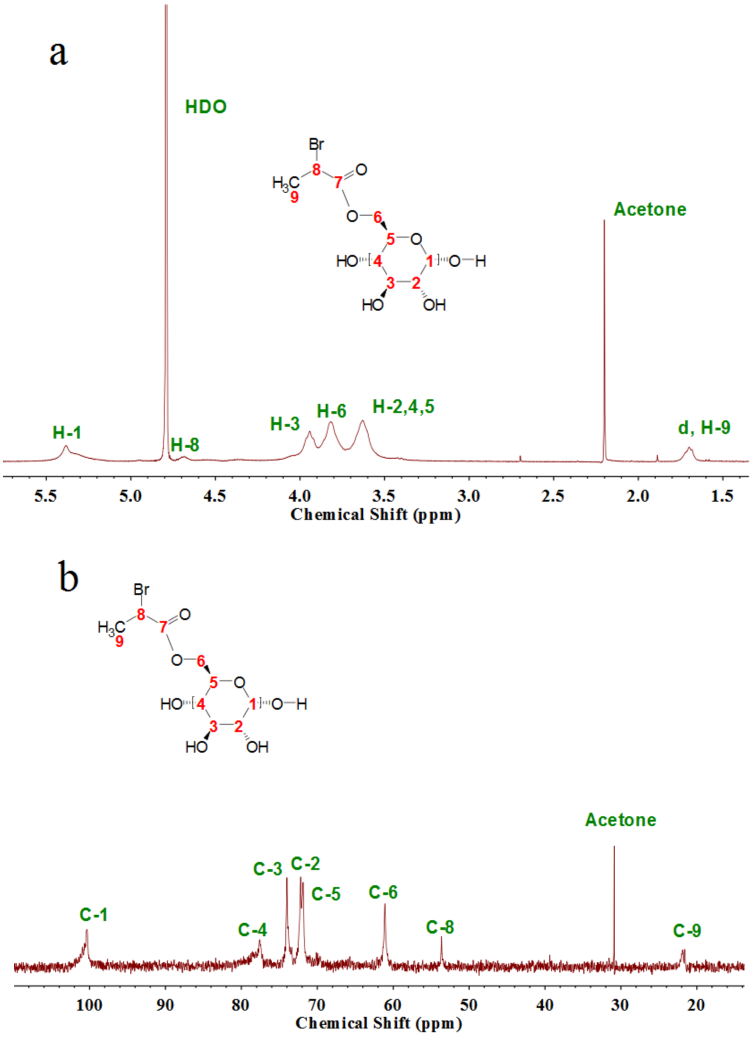

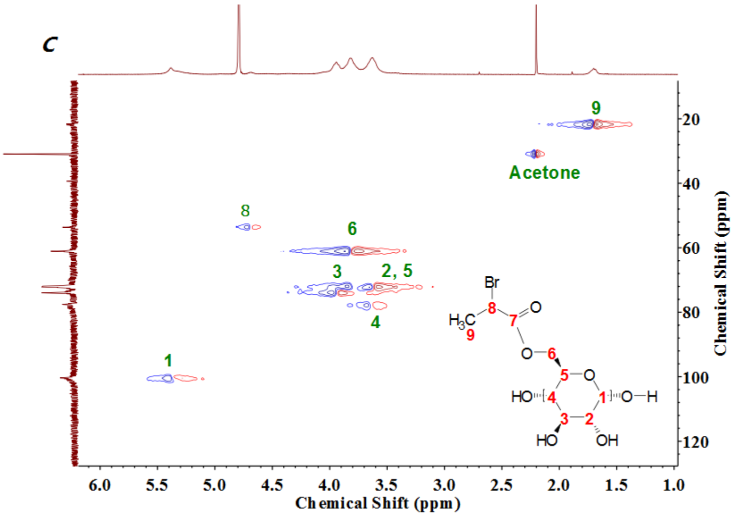

Figure S3. ^{1}H-NMR (**a**), ^{13}C-NMR, (**b**) and gHSQC (**c**) spectra of St-Br (DS = 0.13) in D_2O.

2. The GPC MWD Profile of Starch-g-PNIPAM-P0 and Corresponding Cleaved Polymer

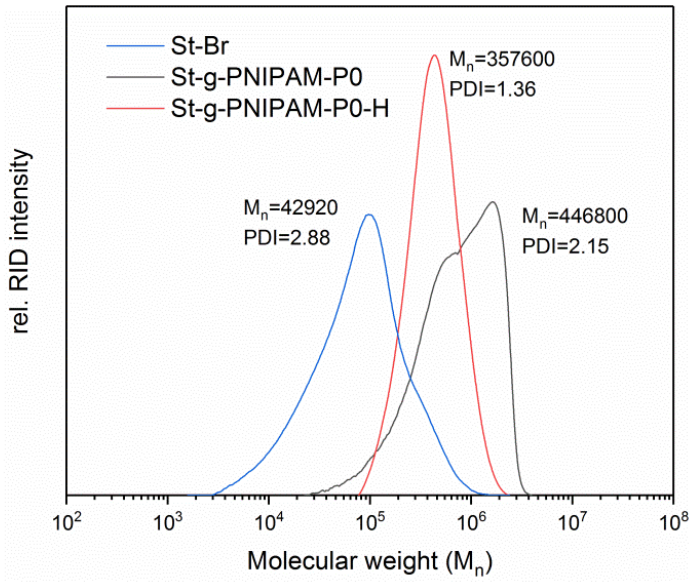

Figure S4. Molecular weight distribution of St-Br, St-g-PNIPAM-P0 before and after hydrolysis (St-g-PNIPAM-P0-H).

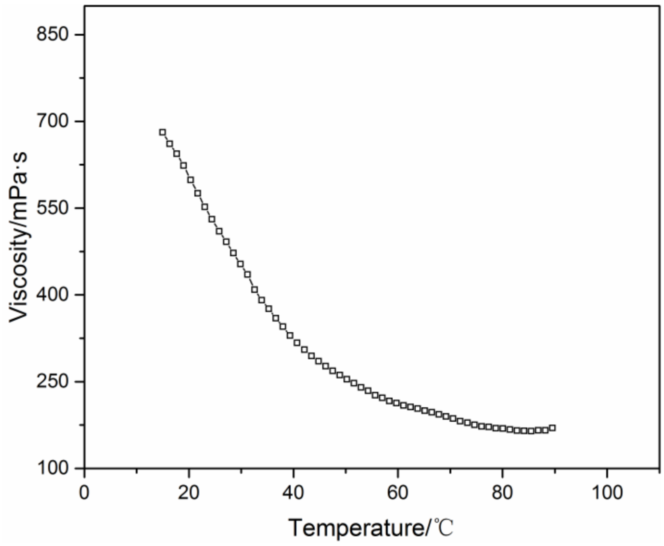

Figure S5. Viscosity versus temperature of PAM (M_n = 35,200) with 1.5 wt % copolymer concentration (γ = 1 s^{-1}).

© 2018 by the authors. Submitted for possible open access publication under the terms and conditions of the Creative Commons Attribution (CC BY) license (http://creativecommons.org/licenses/by/4.0/).
